# 3D-Bioprinting of Stromal Vascular Fraction for Gastrointestinal Regeneration

**DOI:** 10.3390/gels11090712

**Published:** 2025-09-04

**Authors:** Giordano Perini, Margherita Montescagli, Giada Di Giulio, Alberto Augello, Valeria Ferrara, Antonio Minopoli, Davide Evangelista, Matteo Marras, Giulia Artemi, Anna Amelia Caretto, Stefano Gentileschi, Dania Nachira, Valerio Pontecorvi, Cristiano Spada, Loredana Gualtieri, Valentina Palmieri, Ivo Boskoski, Marco De Spirito, Massimiliano Papi

**Affiliations:** 1 Dipartimento di Neuroscienze, Università Cattolica del Sacro Cuore, Largo Francesco Vito 1, 00168 Rome, Italy; giordano.perini@unicatt.it (G.P.); antonio.minopoli@unicatt.it (A.M.); giulia.artemi@unicatt.it (G.A.); 2 UOC Fisica per le Scienze della Vita, Fondazione Policlinico Universitario A. Gemelli IRCSS, 00168 Rome, Italy; alberto.augello@policlinicogemelli.it (A.A.); davide.evangelista@guest.policlinicogemelli.it (D.E.); matteo.marras@guest.policlinicogemelli.it (M.M.); 3 Istituto dei Sistemi Complessi, Consiglio Nazionale delle Ricerche (CNR), Via dei Taurini 19, 00185 Rome, Italy; margheritamontescagli@cnr.it (M.M.); giadadigiulio@cnr.it (G.D.G.); valentina.palmieri@cnr.it (V.P.); 4 Plastic and Reconstructive Surgery, Fondazione Policlinico Universitario Agostino Gemelli IRCCS, 00168 Rome, Italy; annaamelia.caretto@guest.policlinicogemelli.it (A.A.C.); stefano.gentileschi@policlinicogemelli.it (S.G.); 5 Thoracic Surgery, Fondazione Policlinico Universitario Agostino Gemelli IRCCS, 00168 Rome, Italy; dania.nachira@policlinicogemelli.it; 6 Digestive Endoscopy Unit, Fondazione Policlinico Universitario Agostino Gemelli IRCCS, 00168 Rome, Italy; valerio.pontecorvi@policlinicogemelli.it (V.P.); cristiano.spada@policlinicogemelli.it (C.S.); ivo.boskoski@policlinicogemelli.it (I.B.); 7 Department of Surgical Sciences, Sapienza University of Rome, 00161 Rome, Italy

**Keywords:** 3D-bioprinting, stromal vascular fraction, regenerative medicine, intestinal regeneration, GelMA

## Abstract

Intestinal disorders such as inflammatory bowel diseases (IBDs), Crohn’s disease, malabsorption syndromes, and gastrointestinal fistulae (GIFs) are often characterized by chronic inflammation, epithelial barrier disruption, impaired stromal remodeling, and defective angiogenesis. These multifactorial alterations hinder tissue repair and contribute to poor clinical outcomes, with limited efficacy from current therapeutic options. Despite recent advances in surgical and endoscopic techniques, current treatment options remain limited and are frequently accompanied by high morbidity and costs. In this context, regenerative medicine offers a promising avenue to support tissue repair and improve patient care Regenerative medicine offers a promising avenue to restore intestinal homeostasis using advanced biomaterials and cell-based therapies. In this study, we developed a 3D-bioprinted model based on patient-derived stromal vascular fraction (SVF) embedded in a GelMA hydrogel, designed to promote intestinal tissue regeneration. To identify the most suitable hydrogel for bioprinting, we initially evaluated the mechanical properties and biocompatibility of four distinct matrices using bone marrow-derived mesenchymal stromal cells (BM-MSCs). Among the tested formulations, GelMA demonstrated optimal support for cell viability, low oxidative stress, and structural stability in physiologically relevant conditions. Based on these results, GelMA was selected for subsequent bioprinting of freshly isolated SVF. The resulting bioprinted constructs enhanced key regenerative processes across multiple compartments. The SVF-laden constructs significantly enhanced intestinal epithelial cell viability and tight junction formation, as shown by increased trans-epithelial electrical resistance (TEER). Co-culture with fibroblasts accelerated wound closure, while endothelial cells exhibited increased tube formation in the presence of SVF. Together with VEGF secretion, indicating strong paracrine and angiogenic effects. By supporting epithelial, stromal, and vascular regeneration, this approach provides a versatile and translational platform for treating a broad spectrum of intestinal pathologies.

## 1. Introduction

Disorders of the intestinal tract, including inflammatory bowel diseases (IBDs) such as Crohn’s disease and ulcerative colitis, malabsorption syndromes, and gastrointestinal fistulae (GIFs), represent a significant clinical burden and are frequently associated with chronic inflammation, epithelial barrier dysfunction, and impaired tissue repair [1,2,3,4,5,6,7,8,9]. These conditions often result in compromised nutrient absorption, systemic inflammation, and poor quality of life [10,11]. Despite advancements in medical and surgical therapies, current treatments remain largely symptomatic, and many patients experience relapses or require invasive interventions [8,9]. One of the central pathophysiological features of these disorders is the disruption of the intestinal epithelial barrier. This barrier normally prevents the translocation of bacteria and inflammatory stimuli, while allowing selective nutrient absorption [12]. In diseases such as IBD or in the context of post-surgical complications like GIFs, this protective interface is compromised due to dysregulated immune responses, persistent oxidative stress, and defective wound healing [13,14]. Moreover, chronic inflammation not only hinders epithelial regeneration but also affects stromal cells and impairs angiogenesis, further delaying the re-establishment of functional tissue homeostasis [15,16].

Regenerative medicine aims to restore the structure and function of damaged tissues using biologically active materials, including cells, scaffolds, and signaling molecules [17,18]. Among the various cell sources investigated for therapeutic applications, the stromal vascular fraction (SVF) derived from adipose tissue has emerged as a promising and accessible option [19]. SVF is a heterogeneous cell population obtained by enzymatic or mechanical processing of lipoaspirates, and it encompasses a variety of progenitor and immune cells with regenerative, immunomodulatory, and angiogenic capabilities [20,21,22]. The cellular composition of SVF is complex and includes adipose-derived stem cells, endothelial progenitor cells, pericytes, T regulatory cells, monocytes/macrophages, and vascular smooth muscle cells [23]. This diverse population enables SVF to exert multifaceted therapeutic effects that are not solely dependent on cell engraftment, but rather on paracrine signaling and modulation of the local microenvironment. Notably, SVF is rich in growth factors and cytokines, which contribute to tissue regeneration, neovascularization, and immune regulation [24]. One of the main advantages of SVF lies in its minimally manipulated and autologous nature, which reduces the risk of immune rejection and regulatory hurdles. Furthermore, SVF can be isolated intraoperatively and reinjected during the same procedure, making it particularly attractive for point-of-care therapies. Its therapeutic potential has been explored in a variety of clinical contexts, including musculoskeletal injuries, chronic wounds, cardiovascular disease, and, more recently, in the treatment of inflammatory and degenerative disorders [25,26,27,28]. Given its unique combination of cellular diversity, paracrine activity, and ease of isolation, SVF represents a versatile tool in the field of regenerative medicine.

In this study, we investigated the regenerative potential of a 3D-bioprinted model of patient-derived SVF on the intestinal epithelium. Recognizing that the bioink formulation plays a critical role in cell viability and function, we first conducted a comparative analysis of different hydrogel matrices to identify the most biocompatible environment for supporting the heterogeneous cell populations present in the SVF [29]. After selecting the most suitable hydrogel, we developed a composite bioink by incorporating freshly isolated SVF into the optimized hydrogel matrix. This bioink was then employed to fabricate 3D-bioprinted constructs designed to stimulate the dynamics of the intestinal epithelial barrier regeneration. The regenerative potential of SVF was assessed in this model by evaluating its ability to promote epithelial integrity, proliferation, and structural organization. Given the essential role of fibroblast activation and directed migration in tissue repair, extracellular matrix remodeling, and epithelial–mesenchymal crosstalk, we next examined the paracrine influence of SVF on fibroblast chemotaxis [30]. This aspect is particularly relevant for wound healing in the intestinal tract, where stromal cell mobilization is crucial for restoring tissue homeostasis. In addition, we explored the angiogenic capacity of the SVF by evaluating its effect on endothelial cell behavior and microvessel formation. Angiogenesis is a pivotal component of any regenerative process, as the establishment of a functional vascular network ensures nutrient delivery, waste removal, and immune surveillance. Through this multifaceted approach, our work provides new insights into the application of bioprinted SVF for soft tissue regeneration, highlighting its potential not only to support epithelial repair, but also to modulate stromal and vascular responses essential for functional tissue restoration.

## 2. Results and Discussion

### 2.1. Mechanical Characterization of Hydrogels

**Figure 1:**
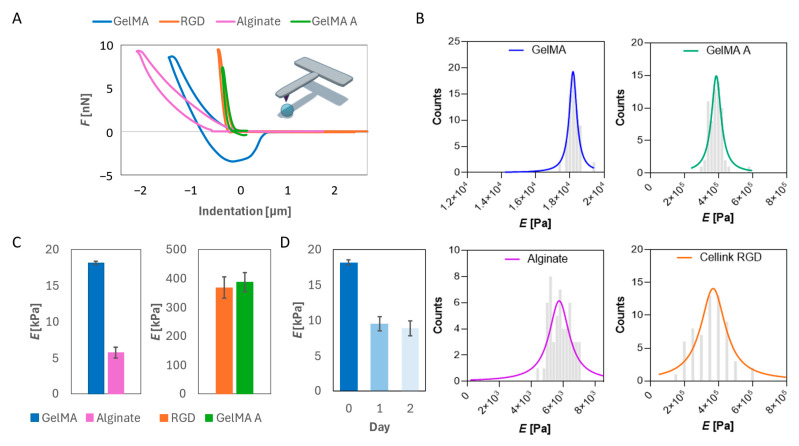
Mechanical characterization of hydrogels. (**A**) Force–indentation curves of the four different hydrogels. (**B**) Frequency distribution of the Young’s Modulus. The histograms represent measured values, while the continuous line represents the Lorentzian fit. (**C**) Peak of the Lorentzian fit for the four different hydrogels. (**D**) Elastic modulus over time of GelMA.

Nanoindentation experiments were performed to evaluate the mechanical properties of four hydrogel formulations: alginate, Cellink-RGD, GelMA, and GelMA A. Representative force–indentation curves (Figure 1A) revealed distinct mechanical behaviors for each material, confirming their diverse viscoelastic responses under loading. Among the tested materials, Cellink-RGD and GelMA A displayed the highest stiffness, with Young’s moduli of 350–400 kPa (Figure 1C), significantly exceeding those of alginate (5.7 ± 0.7 kPa) and GelMA (18.2 ± 0.3 kPa). These values were extracted from the peak of the Lorentzian fit applied to the frequency distributions of individual nanoindentation measurements (Figure 1B). Interestingly, the moduli for Cellink-RGD and GelMA A were markedly higher, reflecting the mechanical reinforcement contributed by nanofibrillated cellulose and the ionic crosslinking of alginate [31,32,33]. Despite their superior stiffness, these formulations may be less suitable for applications requiring a close mimicry of native soft tissue mechanics.

From a biomedical perspective, the mechanical properties of GelMA are particularly relevant. Its stiffness falls within the lower range of native soft tissues such as the brain, liver, and tumor microenvironments, which typically exhibit moduli in the 1–30 kPa range [34]. This makes GelMA an ideal candidate for tissue engineering applications, especially where mechanical cues influence cell fate, migration, or differentiation. Its photo-crosslinkable nature further enhances its versatility for use in 3D bioprinting. Importantly, we noticed an increasing compliance of GelMA over 48 h of approximately 52% due to water absorption (Figure 1D).

### 2.2. Biocompatibility of Hydrogels After Bioprinting

**Figure 2:**
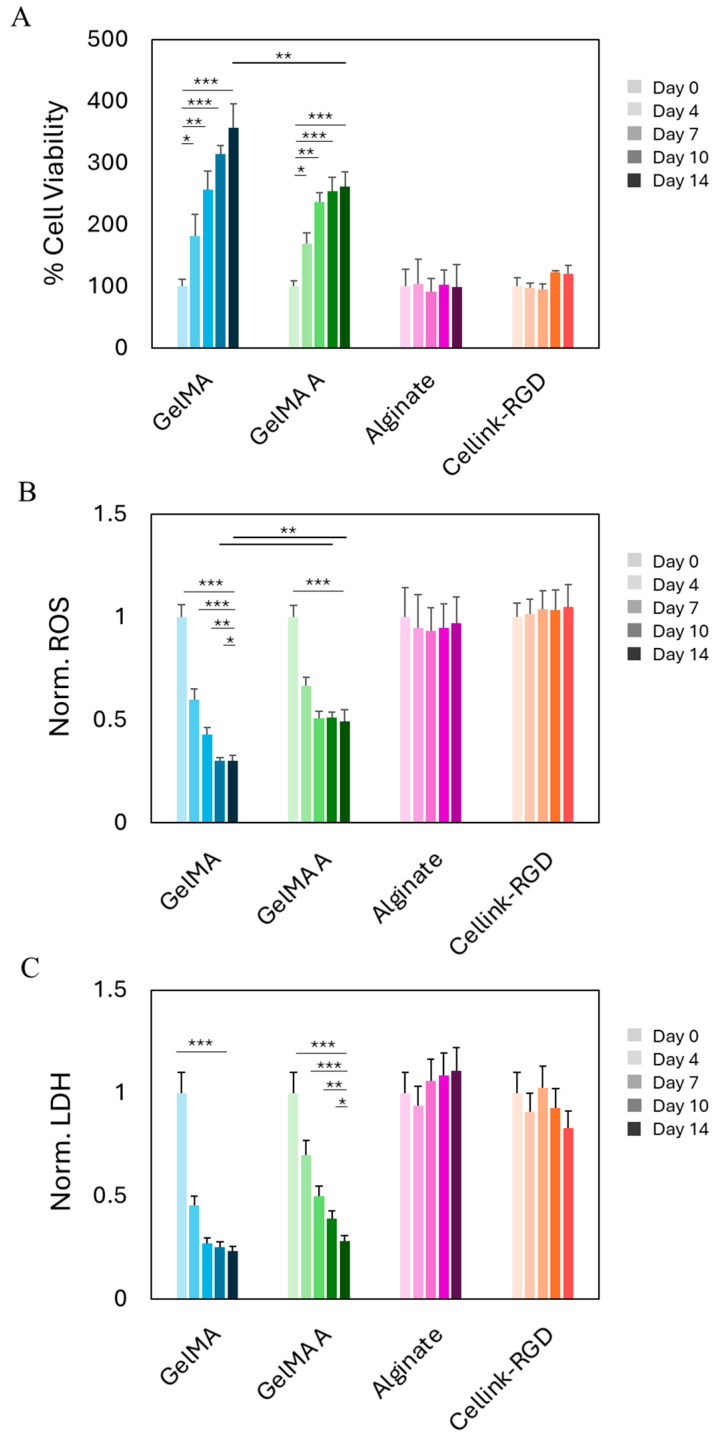
Biocompatibility of hydrogels on BM-MSCs. (**A**) Cell viability over time of BM-MSCs bioprinted with GelMA, GelMA A, Alginate, and Cellink-RGD. Results are expressed as % of day 0 for each bioprinted model. (**B**) Production of ROS over time for BM-MSCs bioprinted with the different hydrogels. Results are normalized by the number of viable cells and reported with respect to day 0 for each hydrogel. (**C**) Cytotoxicity of the four different hydrogels on BM-MSCs over time. Results are normalized by day 0 for each model. *** *p* < 0.001; ** *p* < 0.01; * *p* < 0.05 one-way ANOVA and Tukey post-hoc test.

Following mechanical characterization of the four hydrogel formulations, we conducted biocompatibility assessment using a 3D-bioprinted model comprising commercially available mesenchymal stromal cells. BM-MSCs were selected as a representative model for early-stage hydrogel screening prior to employing SVF, due to their shared mesenchymal phenotype and comparable behavior in terms of adhesion, viability, and matrix interaction. Their relative homogeneity and widespread availability make them well-suited for evaluating the general cytocompatibility of biomaterials in a reproducible and controlled manner. BM-MSCs were bioprinted at a density of 2 × 10^6^ cells/mL into four different hydrogels: alginate, Cellink-RGD, GelMA, and GelMA A. Cell viability was assessed at multiple time points over a 14-day period (Figure 2A). Among the tested formulations, GelMA exhibited the most favorable biological profile, with a significant and sustained increase in normalized cell viability. Cell metabolic activity increased progressively from day 0 to day 14, indicating that GelMA supports both cell survival and proliferation in a 3D-bioprinted environment. GelMA A also showed a statistically significant increase in viability, though to a lesser extent than standard GelMA. This suggests that, despite its chemical modification, GelMA A retains good cytocompatibility and may be appropriate for applications requiring slightly stiffer or functionally tailored matrices. In contrast, Cellink-RGD and alginate did not show significant changes in viability over the 14-day period.

To further characterize cellular response within the various hydrogel matrices, we evaluated oxidative stress and membrane integrity by measuring intracellular ROS levels and LDH release, respectively (Figure 2B,C). All values were normalized to day 0 for each hydrogel condition. ROS levels significantly decreased over time in both GelMA and GelMA A. GelMA showed the greatest reduction, suggesting a reduced oxidative burden and a more supportive microenvironment. Conversely, ROS levels in alginate and Cellink-RGD remained high and stable, indicating persistent oxidative stress likely due to suboptimal matrix–cell interactions or limited diffusivity. Similarly, LDH release decreased markedly in GelMA and GelMA A over time, consistent with improved membrane integrity and reduced cytotoxicity. GelMA again showed the most pronounced effect. In contrast, alginate and Cellink-RGD maintained elevated LDH levels throughout the 14-day culture, suggesting persistent cell membrane damage or impaired recovery following the bioprinting process. Taken together, these findings confirm and expand upon the viability results, demonstrating that GelMA (and to a slightly lesser extent, GelMA A) effectively support cell proliferation while minimizing oxidative stress and cytotoxicity. These characteristics identify them as the most promising candidates among the tested hydrogels for future SVF bioprinting applications.

### 2.3. Growth and Stability of Bioprinted SVF

**Figure 3:**
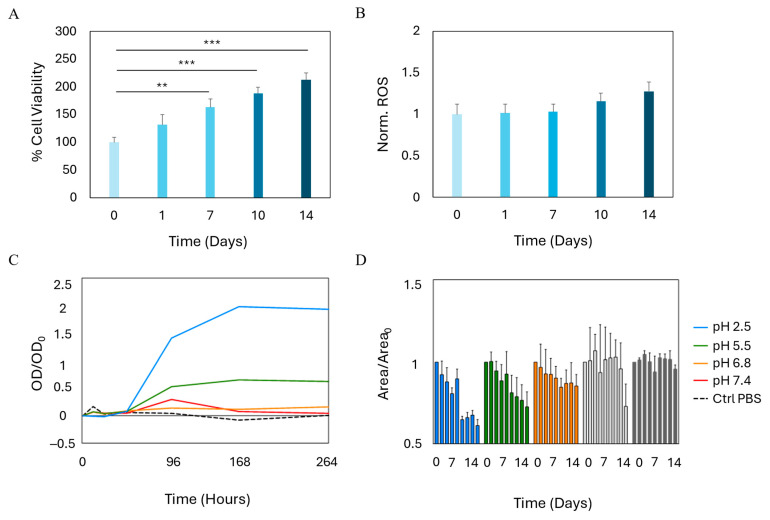
Growth and stability of bioprinted SVF. (**A**) Viability of SVF bioprinted in GelMA over time. Results are expressed as % of day 0. (**B**) Production of reactive oxygen species over time from SVF. Results are normalized to day 0. (**C**) OD variation over 14 days. Results are reported with respect to the initial OD. (**D**) Area variation over 14 days. Results are reported with respect to the initial area of each bioprinted model ± SD. *** *p* < 0.001; ** *p* < 0.01 one-way ANOVA and Tukey post-hoc test.

Following the identification of GelMA as the hydrogel exhibiting the highest degree of biocompatibility among the materials tested, we proceeded with the bioprinting of the SVF. A bioink was formulated by combining GelMA with freshly isolated SVF, and the resulting mixture was bioprinted and crosslinked using the same protocol previously optimized for BM-MSCs. To evaluate the biological performance of the bioprinted constructs, we longitudinally assessed the state of the cellular component in terms of biocompatibility and cytotoxicity. The corresponding results are presented in Figure 3. Notably, cells encapsulated within the GelMA-based constructs exhibited a progressive increase in viability over a two-week culture period, when compared to baseline measurements obtained at day 0 (Figure 3A). This enhancement in cellular viability reached statistical significance beginning on day 7 and became even more pronounced by the end of the second week, indicating a supportive microenvironment conducive to cellular survival and proliferation. Nevertheless, an important consideration for clinical translation of SVF-based therapies involves the potential for elevated ROS production, which can also occur during adipogenic differentiation [35,36]. We therefore investigated the temporal dynamics of ROS levels following bioprinting (Figure 3B). While a slight increase in free radical accumulation was observed starting around day 10, the change was not statistically significant, suggesting that the bioprinting process and the GelMA matrix do not induce substantial oxidative stress—other than an ormetic level—on SVF.

Another key element that should be taken into account for clinical translation is the long-term stability of bioprinted scaffolds. We, therefore, were interested in evaluating the rate of degradation of GelMA bioprinted with SVF at physiological pH. Results are shown in Figure 3C,D. Greater stability was observed under neutral pH conditions (6.8–7.4), where degradation was significantly slowed, preserving the material’s functionality for a longer duration. In contrast, under more acidic conditions (pH 2.5–5.5), a marked increase in degradation rate was detected, as shown by the rapid rise in optical density (Figure 3C), indicative of the release of degradation products into the solution. Importantly, SVF comprises heterogeneous cellular subpopulations, each potentially contributing differently to stability and regenerative outcomes. The behavior and stability of specific subpopulations could be altered in dynamic and tissue-specific microenvironments, particularly in response to biomaterial degradation and remodeling processes.

This behavior can be attributed to hydrolytic degradation, which is amplified in acidic environments, leading to more efficient cleavage of the peptide bonds within the protein chains present in the crosslinked network [37]. This mechanism accelerates the deterioration of the material’s three-dimensional structure.

Simultaneously, a faster reduction in scaffold area was observed at lower pH values (Figure 3D), further supporting the hypothesis of a direct correlation between the acidity of the anatomical site and the degradation rate. This aspect is particularly relevant in pathological contexts, such as fistulas, where the local microenvironment typically exhibits lower pH values compared to healthy tissue [38,39]. Despite these variations, all analyzed scaffolds exhibited a lifespan exceeding 14 days under the tested conditions, demonstrating that the material maintains adequate stability even in acidic environments under in vitro conditions. These findings strongly suggest that the bioprinted SVF is embedded within a hydrogel matrix capable of providing effective trophic support, thereby sustaining cell viability and enabling continuous proliferation over time. These results highlight the potential of this approach as a promising platform for translational regenerative medicine applications and underscore its relevance in a clinical context.

### 2.4. SVF-Enhanced Intestinal Epithelial Regeneration

**Figure 4:**
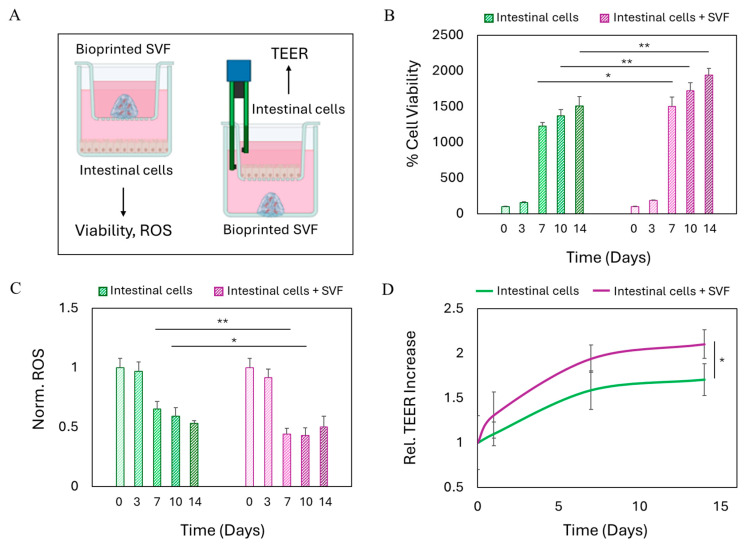
Intestinal epithelial regeneration stimulated by SVF. (**A**) Schematic representation of the experimental setup carried out for the viability of epithelial cells and for intestinal barrier formation. (**B**) Cell viability of intestinal cells over time with or without the presence of SVF. Results are expressed as % of day 0. (**C**) Production of reactive oxygen species over time from intestinal cells with or without the presence of SVF. Results are normalized by the number of viable cells and reported with respect to day 0. (**D**) Increase in the electrical resistance of the intestinal barrier over time. Results are reported with respect to day 0 of control (untreated) cells. ** *p* < 0.01; * *p* < 0.05 one-way ANOVA and Tukey post-hoc test.

Maintaining the integrity of the intestinal barrier is of critical importance for preserving tissue homeostasis and preventing the translocation of luminal contents, including bacteria and pro-inflammatory mediators, into the underlying mucosa and systemic circulation [11,12]. Disruption of this barrier is a hallmark of various gastrointestinal disorders, including inflammatory bowel disease and fistulae, and is often associated with increased oxidative stress, chronic inflammation, and impaired healing. Therefore, strategies aimed at promoting epithelial regeneration and re-establishing barrier function hold substantial therapeutic relevance, particularly in the context of gastrointestinal tissue damage. We therefore designed an experimental model to simulate the clinical scenario of epithelial damage, with the aim of evaluating the potential of bioprinted SVF to promote epithelial recovery and functional barrier restoration. In this setup, human intestinal epithelial cells were seeded onto a transwell membrane to simulate the intestinal barrier, while the 3D-bioprinted SVF construct was positioned on the other compartment. This configuration allowed us to assess the effects of the SVF-laden hydrogel on epithelial cell behavior across a physiologically relevant interface. We monitored two key indicators of intestinal epithelial regeneration over time: (1) the viability and oxidative stress profile of the intestinal layer, and (2) the evolution of TEER, a measure of epithelial barrier integrity and tight junction function. Schematic representation of the experimental setup for the two indicators is reported in Figure 4A. When intestinal epithelial cells were co-cultured with the bioprinted SVF, a significant increase in epithelial cell viability was observed over time, in comparison to untreated controls (Figure 4B). This enhancement suggests that the SVF exerts a trophic effect on the intestinal epithelium, supporting cell survival and proliferation. Moreover, the presence of SVF resulted in a marked reduction in ROS levels within the epithelial monolayer (Figure 4C). Given that oxidative stress is a critical driver of epithelial damage and barrier dysfunction in gastrointestinal pathologies, the observed antioxidant effect of SVF plays a central role in its regenerative activity. The reduction in ROS levels indicates not only a protective effect against oxidative injury but also the creation of a more favorable microenvironment for tissue repair. In parallel, we assessed the functional restoration of the epithelial barrier by monitoring TEER over time (Figure 4D). TEER values progressively increased in the SVF-treated group, suggesting enhanced formation of tight junctions and improved barrier integrity. Notably, the difference in TEER between treated and untreated conditions became statistically significant after 2 weeks, indicating that the presence of SVF actively accelerates the re-establishment of epithelial barrier function. While TEER measurements provided a quantitative and functional assessment of epithelial barrier integrity, we acknowledge that additional analysis of tight junction proteins such as ZO-1 or occludin through immunostaining would further strengthen these findings. Future studies will include molecular confirmation of tight junction formation to complement the observed TEER improvements [40,41]. Nevertheless, the observed improvements in cell viability, oxidative stress, and TEER reinforce the hypothesis that SVF not only supports epithelial cell homeostasis but also contributes to the restoration of tissue functionality, highlighting its potential for therapeutic applications in gastrointestinal disorders characterized by epithelial disruption.

### 2.5. Wound Healing

**Figure 5:**
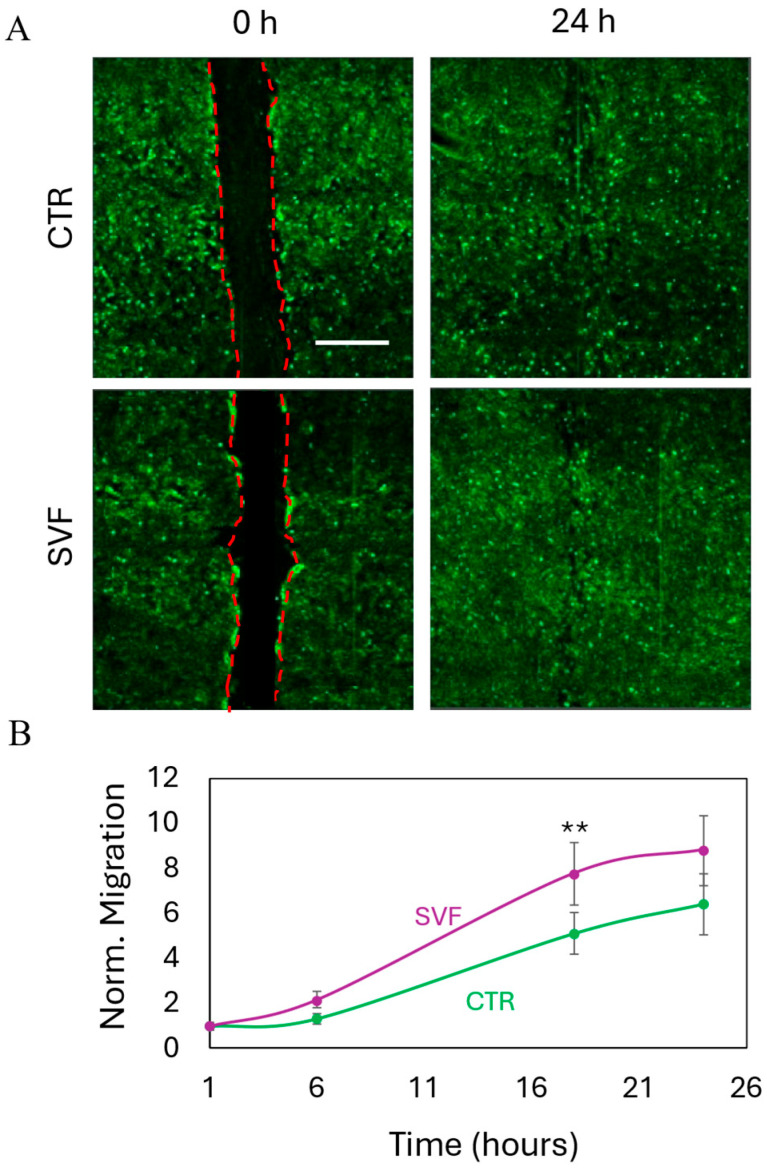
Stimulation of wound healing through bioprinted SVF. (**A**) Images of wound closure at 0 and 24 h. Scalebar 250 μm. (**B**) Quantitative analysis of the migration of fibroblasts toward the wound region over time. Results are normalized to 1 h for both conditions. ** *p* < 0.01 one-way ANOVA and Tukey post-hoc test.

Wound healing represents a critical component of intestinal tissue regeneration, where epithelial and stromal discontinuities impair barrier function and promote chronic inflammation [42]. Fibroblast migration plays a pivotal role in the early phases of wound repair, facilitating extracellular matrix remodeling, paracrine signaling, and tissue remodeling [43]. Evaluating fibroblast chemotaxis in response to bioprinted SVF therefore offers important insights into the pro-regenerative capacity of the construct and its translational potential in treating complex intestinal lesions [42]. To assess this aspect, we conducted a wound healing assay using a transwell system in which a confluent monolayer of human fibroblasts was mechanically scratched to simulate a wound. Bioprinted SVF was positioned in the upper chamber to evaluate its effect on fibroblast migration. Bright-field microscopy was employed to monitor cell behavior at 0 and 24 h post-scratch (Figure 5A). In both SVF-treated and control conditions, fibroblasts migrated to close the wound within 24 h. However, quantitative analysis revealed that the presence of SVF significantly accelerated the closure rate (Figure 5B), indicating a robust chemotactic response. This enhancement of wound closure further supports the multifunctional therapeutic profile of SVF, which not only contributes to epithelial regeneration and angiogenesis but also stimulates the fibroblast-mediated remodeling necessary for durable tissue repair. The integration of these effects underscores the translational relevance of SVF bioprinting as a comprehensive strategy for intestinal healing in challenging pathological settings.

### 2.6. Tube Formation Assay

**Figure 6:**
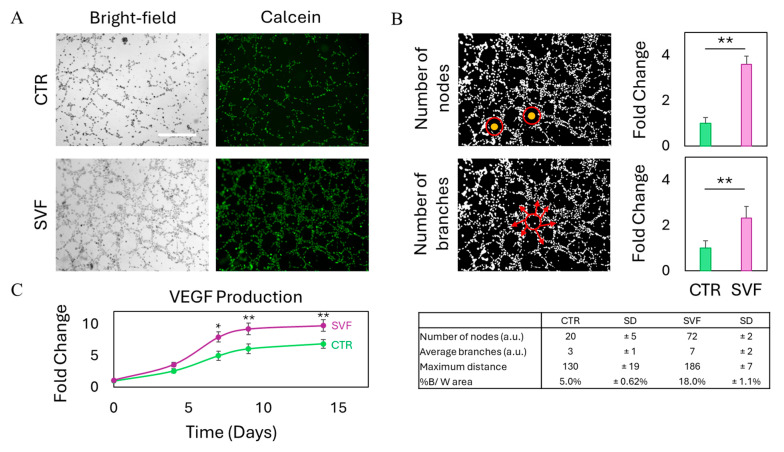
Angiogenesis augmented by bioprinted SVF. (**A**) Bright-field and fluorescence images of the tube formation assay for endothelial cells in the presence of SVF. Scalebar 300 μm. (**B**) Quantitative analysis of tube formation in endothelial cells with and without SVF. Examples of nodes are represented with yellow dots surrounded by red circles, while examples of branches are represented by red arrows. (**C**) Expression of VEGF over time with or without SVF. * *p* < 0.05 and ** *p* < 0.01 one-way ANOVA and Tukey post-hoc test.

The formation of new blood vessels is a critical component of any regenerative process, particularly within the gastrointestinal tract, where efficient tissue repair depends on the timely restoration of microvascular networks [44,45,46]. Angiogenesis ensures the delivery of oxygen and nutrients, removal of metabolic waste, and facilitates immune surveillance, all of which are essential for sustaining epithelial integrity and promoting functional recovery [47]. Impaired vascularization can hinder wound healing and exacerbate inflammation, thereby prolonging tissue damage and delaying closure. Given the heterogeneous cellular composition of SVF, which includes endothelial progenitor cells and pericytes with known pro-angiogenic activity, we sought to assess the ability of SVF to stimulate endothelial tube formation in vitro. To this end, we performed a Matrigel-based tube formation assay on endothelial cells either cultured alone (control) or in the presence of bioprinted SVF constructs. We then conducted a morphological evaluation of tubular structures after waiting overnight to visualize cell viability and network architecture. Results are reported in Figure 6. When compared to the control condition, in which endothelial cells were cultured alone, the co-culture with bioprinted SVF constructs led to a marked increase in the formation of capillary-like tubular structures, as observed in both bright-field and calcein-stained fluorescence images (Figure 6A). The network formed in the presence of SVF appeared more extensive and interconnected, suggesting enhanced endothelial activation and organization. Quantitative analysis confirmed these morphological observations, showing a statistically significant increase in key angiogenic parameters such as total tube length, number of branching points, and mesh formation in the SVF-treated group (Figure 6B). This stimulatory effect is likely attributable to the secretion of pro-angiogenic factors by SVF, particularly VEGF, which plays a central role in endothelial cell proliferation, migration, and capillary morphogenesis. Accordingly, we measured an upregulation of VEGF expression over time in the SVF-treated condition, further supporting the hypothesis of a paracrine-driven mechanism (Figure 6C). In the context of gastrointestinal epithelial repair, such angiogenic stimulation is particularly relevant, as the formation of a functional microvasculature not only facilitates nutrient and oxygen delivery but also contributes to the resolution of inflammation and the re-establishment of tissue homeostasis. The integration of angiogenic capacity with epithelial and stromal support further highlights the multifactorial regenerative potential of SVF bioprinted constructs, strengthening their translational appeal for the treatment of complex intestinal pathologies.

## 3. Conclusions

In this study, we demonstrated the feasibility and therapeutic relevance of a bioprinted SVF model for the regeneration of intestinal epithelial tissue. By systematically evaluating multiple hydrogel formulations, we identified GelMA as the most suitable matrix to support the viability, proliferation, and functional stability of SVF-derived cellular constructs. The GelMA-based bioink not only provided a biocompatible environment for SVF, but also ensured scaffold durability under physiologically relevant pH conditions, including those mimicking the microenvironments often found in the gastrointestinal tract. Our in vitro co-culture models revealed that bioprinted SVF promotes key regenerative responses across multiple cellular compartments. Specifically, bioprinted SVF enhanced epithelial cell viability, reduced oxidative stress, and accelerated the re-establishment of barrier integrity, as evidenced by increased trans-epithelial electrical resistance. Moreover, SVF stimulated fibroblast migration in a wound healing assay and significantly increased angiogenic activity in endothelial cells, with upregulation of VEGF expression and the formation of complex vascular networks. Taken together, these findings highlight the multifactorial regenerative potential of SVF when delivered in a bioprinted format, supporting not only epithelial regeneration, but also stromal and vascular remodeling. This approach offers a promising platform for translational applications in gastrointestinal tissue repair and opens new perspectives for the development of advanced, patient-derived therapies for conditions such as intestinal fistulae.

## 4. Materials and Methods

### 4.1. Hydrogel Preparation

GelMA, GelMA A, Cellink-RGD, and alginate were obtained from Cellink (CELLINK). For each condition, 20 µL of hydrogel was dispensed onto a Petri dish. Alginate and Cellink-RGD (alginate covalently conjugated with the RGD peptide for cell adhesion and reinforced with nanofibrillated cellulose) were crosslinked using a 3% (*w*/*v*) CaCl_2_ solution for 5 min. GelMA (gelatin methacrylated) was crosslinked with UV light (405 nm) for 1 min. GelMA A, a composite blend of GelMA and alginate, was crosslinked sequentially using both crosslinking methods. Following crosslinking, all samples were incubated in sterile water at room temperature overnight.

### 4.2. Atomic Force Microscopy

Atomic force microscopy (AFM) indentation experiments were conducted using a standard setup where a cantilever with a specifically shaped tip approaches the sample surface at a controlled speed. The cantilever indents the sample until a predefined maximum force is reached, then retracts. Throughout the process, cantilever deflection and displacement are recorded and used to assess the mechanical properties of the sample. The Young’s modulus is typically extracted from the resulting force–indentation curves [31]. In this study, measurements were performed using a Nanowizard II AFM system (JPK Instruments) equipped with HQ:CSC37/No Al tips (MikroMasch, Tallin, Estonia), featuring a nominal spring constant of 0.3 N/m, a tip radius of 8 nm, and a cone angle of 40°. The spring constant was calibrated via thermal tuning prior to each experiment. Force–indentation curves were recorded using a force limit of 5.5 nN and an indentation speed of 15 µm/s, with a maximum indentation depth of 2 µm. For each condition, 64 measurements were performed in a 10 µm × 10 µm grid. Young’s modulus values were determined using the JPK Data Processing software (6.1 version) by fitting each force curve with the Hertz-Sneddon model, assuming a Poisson’s ratio of 0.33. The resulting frequency distributions were fitted with Lorentzian functions, and the peak of each distribution was used to represent the Young’s modulus.

### 4.3. Cell Culture

Human bone marrow mesenchymal stem cells (BM-MSCs), Caco-2 human intestinal epithelial cells, BJ human fibroblasts, and EA.hy926 human endothelial cells were purchased from the American Type Culture Collection (ATCC). BM-MSCs were cultured in Mesencult (STEMCELL, Vancouver, Canada) supplemented with 1% penicillin–streptomycin (Sigma-Aldrich, St. Louis, MO, USA). Caco-2 cells were cultured in DMEM (Sigma-Aldrich) supplemented with 20% FBS (EuroClone, Milan, Italy) and 1% penicillin–streptomycin. BJ fibroblasts were cultured in DMEM and M199 (Euroclone) at a ratio of 4:1, respectively, supplemented with 10% FBS and 0.02% igromicine (Sigma-Aldrich). EA.hy926 were cultured in DMEM supplemented with 10% FBS and 1% penicillin–streptomycin. Cells were kept in T75 flasks (Corning, NY, USA) at 37 °C under 5% CO_2_ for further treatments.

### 4.4. Isolation of SVF

The harvested fat, taken from the medial region of the thighs through a suction microcannula, necessitates the use of a Digital Vortex Mixer (Elea Surgere S.R.L., Verona, Italy). After placing the syringes with the lipoaspirate contents in the disposable cup on the Vortex Mixer, an orbital force of 2000 rpm is applied for 8 min. This process activates the atMSCs and separates the lipidic part and the serum. The liquid and the oil are discharged; instead, the remaining fat is emulsified, passing through a 2 mm filter and then loaded in a 19-gauge endoscopic needle for the endoscopic injection in the submucosal layer around the fistula.

### 4.5. Bioprinting of BM-MSCs and SVF

To create the bioink for bioprinting BM-MSCs, cells at a density of 2 × 10^6^ cells/well were mixed with the four different hydrogels at a ratio of 2:1, respectively, by using a double-syringe system connected with a luer-lock adaptor. The same procedure was carried out for SVF, which was mixed at a ratio of 2:1 with GelMA.

The bioinks were then loaded on a 3 mL bioprinting cartridge (CELLINK, Boston, MA, USA). The cartridge was then transferred to a Bio X6 bioprinter (CELLINK). A droplet print of the formed bioink was used as a printing strategy. A temperature controlled printhead (CELLINK) was mounted on the BIO X6, using a nozzle diameter of 18 G (840 µm). The printbed temperature was set at RT, while the printhead was set at 30 °C. Bioprinting was performed by extruding at 10 kPa, with an extrusion time of 2 s on a 24-well plate (Corning). After droplet extrusion, photocrosslinking was performed with a photocuring printhead (CELLINK) at a distance of 3 cm from the samples, for 30 s. Fresh culture medium was added to each bioprinted model, and the plate was incubated at 37 °C, 5% CO_2_ for further experiments.

### 4.6. Degradation of Bioprinted SVF

Bioprinted models were subjected to degradation testing by immersion in phosphate-buffered saline (PBS, Corning) solutions with different pH values, selected to simulate the main anatomical regions where intestinal discontinuities commonly form. The following pH values were used: pH 2.5 to mimic the gastric environment, pH 5.5 for the proximal colon, pH 6.8 for the distal colon/rectum, and pH 7.4 for the small intestine. Models were incubated in PBS solutions at 37 °C for 14 days. During this period, both the optical density (OD) of the solution and the dimensional changes of the samples were monitored at specific time points: 0 h, 1 h, 12 h, 1 day, 2, 4, 7, 11, and 14 days. For OD measurements, the reference parameter was the ratio between the OD at time zero and that measured at each time point. For sample dimensions, the reference parameter was the ratio between the initial area and the area at the corresponding time point.

### 4.7. Cell Viability

To test cell viability on bioprinted BM-MSCs over time on the four different hydrogels, we used Real Time-Glo^®^ MT Cell Viability Assay (Promega, Madison, WI, USA) following the manufacturer’s instructions. Briefly, MT Cell Viability Substrate and NanoLuc^®^ Enzyme were diluted in culture medium at a final concentration of 2×, to obtain the RealTime-Glo™ working solution. An amount of 2× RealTime-Glo™ working solution equal to the volume of culture medium was added to each well. The plate was then incubated at 37 °C, 5% CO_2_ for an hour. Luminescence was recorded with Cytation 3 Cell Imaging Multi-Mode Reader (Biotek, Winooski, VT, USA). To test viability over time on SVF bioprinted with GelMA, we used Real Time-Glo^®^ MT Cell Viability Assay as described above. Data were reported as % of day 0. To test viability on the Caco-2 cell line in a timespan of 2 weeks, we seeded cells at a concentration of 2 × 10^4^ cells/well on the bottom chamber of a 24-well transwell system (Corning), having a pore size of 5 µm. We then transferred bioprinted SVF models onto the upper chamber of the transwell system. We monitored cell viability of Caco-2 by using Real Time-Glo^®^ MT Cell Viability Assay as described above.

### 4.8. Trans-Epithelial Electrical Resistance

Trans-epithelial electrical resistance (TEER) was monitored on Caco-2 cells to observe intestinal epithelial layer formation. For this purpose, we seeded Caco-2 cells on the top chamber of a transwell system having pores of 0.4 μm. Bioprinted SVF was then put in the bottom chamber of the transwell. TEER was measured by using a Millicel^®^ ERS-2 electrical resistance system (Millipore, Darmstadt, Germany). To properly measure TEER, a blank transwell filled with culture medium was used. Recorded blank values were then subtracted from experimental values of Caco-2, and the resulting Ω were multiplied by the area of the transwell insert. Results are then reported as fold change of day 0 for both Caco-2 cells with and without bioprinted SVF.

### 4.9. Reactive Oxygen Species Production

Production of reactive oxygen species (ROS) was measured on BM-MSCs bioprinted with the four different hydrogels and on SVF bioprinted with GelMA by using a fluorinated derivative of 2′,7′-di-chlorofluorescein (H_2_DCFDA, Invitrogen). This probe is initially non-fluorescent and becomes fluorescent only after intracellular esterases cleave its acetate groups, allowing subsequent oxidation to occur within the cell. Oxidation events were therefore tracked by measuring the rise in fluorescence intensity. At each timepoint, culture medium was replaced with PBS containing 10 μM H_2_DCFDA. Bioprinted models were incubated for 30 min at 37 °C in 5% CO_2_. Solution was then removed, and bioprinted models were resuspended in complete medium. The fluorescence intensity of H_2_DCFDA was recorded by using a Cytation 3 Cell Imaging Multi-Mode Reader using an excitation wavelength of 495 nm and recording the emission at 528 nm. To measure ROS production over time of Caco-2 cells at a concentration of 2 × 10^4^ cells/well on the bottom chamber of a 24-well transwell system, having a pore size of 5 µm. We then administered H_2_DCFDA following the procedure described above. Results were normalized by the number of viable cells and reported as a fold change of day 0.

### 4.10. Lactate Dehydrogenase Release

To measure cytotoxicity on BM-MSCs, we used a lactate dehydrogenase (LDH, Roche, Basel, Switzerland) assay. Briefly, the LDH was evaluated on the supernatants of bioprinted models. Each supernatant was diluted before incubation with the substrate. Thirty minutes later, absorbance at 450 nm was measured with a Cytation 3 Cell Imaging Multi-Mode Reader. Results were normalized by the number of viable cells and reported with respect to day 0.

### 4.11. Wound Healing Assay

BJ cells were seeded at a concentration of 1.8 × 10^5^ cells/well on the bottom chamber of a 24-well transwell system, having a pore size of 5 µm. Cells were maintained at 37 °C, 5% CO_2_ for 24 h to allow cell adhesion and formation of a confluent monolayer. Cells were then scored with a sterile pipette tip to leave a scratch of ~0.3 mm in width. Culture medium was removed, and wells were washed with PBS to remove dislodged cells. The removed medium was replaced with fresh culture medium. We then transferred bioprinted SVF onto the upper chamber of the transwell system. The plate was incubated at 37 °C, 5% CO_2_, and bright-field images were acquired over time with Cytation 3 Cell Imaging Multi-Mode Reader to observe wound closure.

### 4.12. Tube Formation Assay

Prior to endothelial cell seeding, the bottom of a transwell 24-well plate was coated with 0.3 mL Matrigel^®^ (Corning) at a concentration of 10 mg/mL. The plate was then incubated at 37 °C for 1 h, and the excess Matrigel was removed. EA.hy926 cell line was seeded on top of coated wells at a concentration of 1.2 × 10^5^ cells/well. We then transferred bioprinted SVF onto the upper chamber of the transwell system having a pore size of 5 µm and incubated overnight at 37 °C, 5% CO_2_. The day after, cells were stained with calcein-AM (Invitrogen, Waltham, MA, USA) following the manufacturer’s procedures. Briefly, calcein-AM at the initial concentration of 1 mg/mL was diluted with PBS at a final concentration of 10 µM, to obtain the final working solution. Medium of the lower chamber was then replaced with calcein working solution and incubated at 37 °C for 20 min. Bright-field and fluorescence images were acquired with the Cytation 3 Cell Imaging Multi-Mode Reader to observe tube formation. Images were analyzed with Fiji (ImageJ version 1.54) software, and key parameters of tube formation were measured. In particular, the number of nodes and branches was analyzed. Nodes refer to the aggregates from which endothelial cells form tubes, while the branches are the completely formed tubes. Results were normalized by control (untreated) endothelial cells.

### 4.13. ELISA Assay

Supernatants were collected, and the concentration of vascular-endothelial growth factor (VEGF) released by SVF was determined by Human VEGF-A ELISA kit (Sigma-Aldrich). The assay was performed on undiluted conditioned media, and each sample was tested in triplicate. According to the manufacturer’s instructions, 100 µL of sample and standard working solution were loaded in a 96-well plate and incubated for 2.5 h at room temperature with gentle shaking. The solution in each well was removed, and the biotinylated detection Ab working solution was added. Following a 1-h incubation, HRP-conjugated streptavidin was added and incubated for an additional 45 min. Between each incubation step, the wells were washed four times with wash buffer. Subsequently, the TMB substrate solution was added and incubated for 30 min at room temperature, protected from light, until the color turned blue. The reaction was stopped by adding 50 µL of stop solution to each well, resulting in a color change to yellow. Absorbance was immediately measured at 450 nm using a Cytation 3 Cell Imaging Multi-Mode Reader.

### 4.14. Statistical Analysis

All measurements were performed in triplicate. Statistical analysis was performed using one-way ANOVA, followed by Tukey’s post-hoc test. Significance was expressed as * *p* < 0.05, ** *p* < 0.01 and *** *p* < 0.001.

## Data Availability

The data presented in this study are available on request from the corresponding author.
